# Response to: “POCUS to Confirm Intubation in a Trauma Setting”

**DOI:** 10.5811/westjem.2020.9.50017

**Published:** 2020-12-16

**Authors:** Michael Gottlieb, Stephen Alerhand, Brit Long

**Affiliations:** * Rush University Medical Center, Department of Emergency Medicine, Chicago, Illinois; † Rutgers New Jersey Medical School, Department of Emergency Medicine, Newark, New Jersey; ‡ Brooke Army Medical Center, Department of Emergency Medicine, San Antonio, Texas

To the Editor:

We thank the authors for their insights and for sharing this case. The authors describe a patient who was intubated with the endotracheal tube (ETT) located at the tip of the carina, thereby allowing for bilateral lung sliding, while placing the ETT at risk of converting to a mainstem intubation.

This case highlights the importance of considering ETT depth and the potential for suboptimal, deep ETT placement to be present even when bilateral lung sliding is present. This can be particularly dangerous among younger patients (who have shorter tracheal lengths) and when there is a high probability of ETT movement, such as in the prehospital setting or when transferring between beds.

We agree with the authors that, while bilateral lung sliding can confirm that mainstem intubation is not present, it is not sufficient to confirm the optimal ETT depth. In these cases, additional assessment strategies, such as numeric depth assessment and direct ETT cuff visualization, offer additional information to supplement lung sliding. Moreover, we believe it is important to consider point-of-care ultrasound (POCUS) as a serial test used to assess initial position and reassess the position when the clinical condition changes or there is concern for ETT movement. This would allow rapid identification of mainstem intubation and reduce the time to intervention compared with radiographs.^1^

We appreciate the authors concern regarding inflating the ETT cuff with saline. However, it can be more challenging to visualize an air-filled ETT cuff, and several studies have assessed the use of a saline-filled ETT cuff to confirm ETT depth without identified complications.^2^,^3^ With regard to transtracheal ultrasound, many cervical collars have a central opening in the anterior aspect which could be used to assess for ETT placement with POCUS using the transtracheal approach. We propose that transtracheal ultrasound is an important aspect of the ETT confirmation technique and can assess for ETT location, as well as hypopharyngeal placement.^1^,^4^,^5^ As the body of literature regarding POCUS for ETT confirmation continues to grow, we believe future research should prospectively assess combined POCUS protocols and identify which approach is best for determining the ideal ETT depth.
